# Fourth Generation Phosphorus-Containing Dendrimers: Prospective Drug and Gene Delivery Carrier

**DOI:** 10.3390/pharmaceutics3030458

**Published:** 2011-08-05

**Authors:** D. Shcharbin, V. Dzmitruk, A. Shakhbazau, N. Goncharova, I. Seviaryn, S. Kosmacheva, M. Potapnev, E. Pedziwiatr-Werbicka, M. Bryszewska, M. Talabaev, A. Chernov, V. Kulchitsky, A.-M. Caminade, J.-P. Majoral

**Affiliations:** 1 Institute of Biophysics and Cell Engineering of Natl. Acad. Sci, Minsk, Belarus; 2 Department of Clinical Neuroscience, Faculty of Medicine, University of Calgary, Calgary, Canada; E-Mail: shakhbazau@gmail.com; 3 Republic Center for Hematology and Transfusiology, Minsk, Belarus; E-Mail: ihar.in@gmail.com (I.S.); 4 Department of General Biophysics, University of Lodz, Lodz, Poland; E-Mail: marbrys@biol.uni.lodz.pl (M.B.); 5 City Hospital of Emergency Help, Minsk, Belarus; 6 Institute of Physiology of Natl. Acad. Sci, Minsk, Belarus; E-Mail: vladi@fizio.bas-net.by (V.K.); 7 Laboratorie de Chimie de Coordination, CNRS, Toulouse, France; E-Mail: majoral@lcc-toulouse.fr.

**Keywords:** phosphorus-containing dendrimer, drug delivery, gene delivery, dendriplex characterization, transfection, mesenchymal stem cells, tumor, cisplatin

## Abstract

Research concerning new targeting delivery systems for pharmacologically active molecules and genetic material is of great importance. The aim of the present study was to investigate the potential of fourth generation (P4) cationic phosphorus-containing dendrimers to bind fluorescent probe 8-anilino-1-naphthalenesulfonate (ANS), anti-neoplastic drug cisplatin, anti-HIV siRNA siP24 and its capability to deliver green fluorescent protein gene (pGFP) into cells. The interaction between P4 and ANS (as the model drug) was investigated. The binding constant and the number of binding centers per one molecule of P4 were determined. In addition, the dendriplex between P4 and anti-HIV siRNA siP24 was characterized using circular dichroism, fluorescence polarization and zeta-potential methods; the average hydrodynamic diameter of the dendriplex was calculated using zeta-size measurements. The efficiency of transfection of pGFP using P4 was determined in HEK293 cells and human mesenchymal stem cells, and the cytotoxicity of the P4-pGFP dendriplex was studied. Furthermore, enhancement of the toxic action of the anti-neoplastic drug cisplatin by P4 dendrimers was estimated. Based on the results, the fourth generation cationic phosphorus-containing dendrimers seem to be a good drug and gene delivery carrier candidate.

## 1. Introduction

Dendrimers are a new class of polymers with a well-defined molecular structure [1-6] that combine defined composition and monodispersity with high molecular mass, resulting in numerous interesting physical and chemical properties. The dendrimer characteristics are as follow: they possess several functional end groups, which are responsible for high solubility and reactivity, and empty internal cavities [1,2]. These properties contribute to the suitability of dendrimers for targeting drugs, nucleic acids and short oligodeoxynucleotides (ODN). Phosphorus-containing fourth generation dendrimers were synthesized in the Laboratoire de Chimie de Coordination du CNRS [3,4]. They are characterized by the presence of aminothiophosphates at each branching point along the backbone (Figure 1), which may enhance biocompatibility [3]; P4, C1296H2256N375Cl96O90P93S90 (generation 4, 96 surface cationic end groups, Mw: 33,702; diameter: 5 nm) [4].

Cationic phosphorus-containing dendrimers reduce replication of the abnormal scrapie isoform of the prion protein in mice [5] and the effects of phosphorus-containing dendrimers on the innate immune system were reported recently [6-8]. Human monocytes can be activated by acid phosphoniccapped, phosphorus-containing dendrimers, as indicated by morphological and phenotypic modifications to the cells, together with an increase in phagocytosis and survival [6,7].

The scope of present study includes investigation of P4 as a binding agent for model and therapeutic molecules as well as the influence of dendrimers and dendriplexes on viability of embryonic cells and mesenchymal stem cells. Efficiency of the dendrimers to convey model a plasmid into cells and release it was shown by transfection experiments.

## 2. Experimental Section

### 2.1. Materials

The sequences of siP24 (siRNA) were: sense, GAUUGUACUGAGAGACAGGCU; antisense: CCUGUCUCUCAGUACAAUCUU (Sigma, USA).

### 2.2. Fluorescence of l-anilinonaphthalene-8-sulfonic acid

The binding constant (*K*_b_) and the number of binding centers per one molecule (*n*) of dendrimers and human serum albumin were determined using a double fluorometric titration technique [9]. In the first fluorometric titration of ANS, increasing concentrations of the binding agent (BA) were added to a constant concentration of ANS and the extreme intensity (*F*_max_) of ANS fluorescence was determined, which corresponded to the state where all ANS molecules were bound by the binding agent. The extreme fluorescence intensity of ANS divided by its concentration gave the specific fluorescence intensity for the bound probe (*F*_sp_):

(1) $${\text{F}}_{\text{sp}}=\frac{{\text{F}}_{\max}}{{\text{C}}_{\text{ANS}}^{1}}$$

where *C*^1^_ANS_ is ANS concentration during the first fluorometric titration.

In the second fluorometric titration, the binding agent had a constant concentration (C_BA_). Increasing concentrations of ANS(*C*_ANS_) were added to the binding agent and the fluorescence intensity (*F*) was measured. The concentration of ANS bound by the binding agent was calculated as:

(2) $${\text{C}}_{\text{ANS}}^{\text{bound}}=\frac{\text{F}}{{\text{F}}_{\text{sp}}}$$

and concentration of free ANS as:

(3) $${\text{C}}_{\text{ANS}}^{\text{free}}={\text{C}}_{\text{ANS}}-{\text{C}}_{\text{ANS}}^{\text{bound}}$$

The binding constant (*K*_b_) and the number of binding centers in solution (*N*) can be determined from the plot of $1/{\text{C}}_{\text{ANS}}^{\text{bound}}$ on the ordinate *versus* $1/{\text{C}}_{\text{ANS}}^{\text{free}}$ on the abscissa, according to the equation:

(4) $$\frac{1}{{\text{C}}_{\text{ANS}}^{\text{bound}}}=\frac{1}{{\text{K}}_{\text{b}}\cdot \text{N}\cdot {\text{C}}_{\text{ANS}}^{\text{free}}}+\frac{1}{\text{N}}$$

The initial region of the curve is a straight line. We modified Eq. (4) by replacing N by the number of binding centers per one molecule of the binding agent (*n*):

(5) $$\text{n}=\frac{\text{N}}{{\text{C}}_{\text{BA}}}$$

where *C*_BA_ is a molar concentration of the binding agent.

Thus, the final version of Eq. (4) was:

(6) $$\frac{{\text{C}}_{\text{BA}}}{{\text{C}}_{\text{ANS}}^{\text{bound}}}=\frac{1}{{\text{K}}_{\text{b}}\cdot \text{n}\cdot {\text{C}}_{\text{ANS}}^{\text{free}}}+\frac{1}{\text{n}}$$

### 2.3. Circular dichroism

The CD spectra of P4/siRNA complexes were measured with a Jasco-815 spectropolarimeter. P4 concentrations used in the experiments were defined by the range 0 to 43.12 μM. Measurements were performed in a buffer of 0.15 M sodium phosphate (pH 7.4) containing 0.1 M NaCl at 25 °C.

Spectra were corrected against the baseline obtained using a dendrimer sample, and were smoothed using a binomial algorithm provided by Jasco. Spectra demonstrate the average of two independent replicates. Scans were obtained from 320 to 200 nm at a rate of 50 nm min^−1^ with a bandwidth of 1 nm in 650 μL quartz cuvettes with a path length of 0.5 cm.

### 2.4. Particle size and zeta potential

Samples intended for light scattering analyses were prepared at 25 °C using 0.15 M PBS (0.05 M phosphate buffer + 0.1 M NaCl), pH 7.4, which was passed through a 0.22 μm filter to remove trace particulates. Complexes were prepared at 0.5 μmol/L siRNA and at molar ratios of P4/siRNA ranging between 0 and 16.

The particle size of complexes was measured using dynamic light scattering (DLS) and a Malvern Zeta-Sizer Nano S90 (Malvern, UK). Light scattered at 90° from the incident light was fitted to an autocorrelation function using the method of cumulants. The particle size of a sample was determined from the average of 12 cycles in a Malvern disposable plastic cuvette at 25 °C.

Zeta potential experiments were carried out using phase analysis light scattering (PALS), and a Malvern Instruments Zetasizer 2000 (Malvern, UK) at 25 °C. The electrophoretic mobility of the scattering (DLS) samples was determined from the average of six cycles of an applied electric field in a standard rectangular quartz cell. The zeta potential of complexes was determined from the electrophoretic mobility using the Smoluchowski approximation.

### 2.5. Ethidium bromide intercalation assay

The fluorescent dye ethidium bromide (EB) can intercalate into double-stranded DNA or RNA. It occupies an effective binding site of several base pairs, leading to a significant increase of its fluorescence intensity and to a blue-shift of its maximum emission wavelength $\left(\lambda_{\max}^{\text{em}}\right)$. Compounds with higher affinity for DNA (e.g. dendrimers) displace the dye, quench its fluorescence and induce a red-shift of its $\lambda_{\max}^{\text{em}}$. EB was added to pGFP solution at a concentration of 1 molecule of dye per 1 bp of DNA and its fluorescence was monitored using a JASCO-FP 6300 spectrofluorimeter (JASCO GmbH, Germany). The excitation wavelength was 477 nm; the excitation and emission slits were 10 nm. The emission spectra were recorded between 500 and 700 nm and the position of emission maximum was determined. The ‘dye-pGFP’ complex was titrated with a dendrimer and changes in the fluorescence parameters (intensity and $\lambda_{\max}^{\text{em}}$) were recorded [10]. Measurements were performed at 25 °C.

### 2.6. Fluorescence polarization of fluorescein

This technique is based on changes of fluorescence polarization of fluorescein labeled siRNA when a dendrimer is added. Labeled ODN in solution at 25 °C is quite flexible. Dendriplex formation leads to significant restrictions of ODN molecular motions and increases the molecular mass of complexes, apparent in a significant increase (up to 4-times) of its degree of fluorescence polarization. The destruction of dendriplex leads to significant decrease of fluorescence polarization of fluorescein labeled siRNA.

### 2.7. Vector construction and preparation

Experiments to study the gene delivery potential of phosphorus-containing dendrimers were performed with the plasmid vector pAAV-IRES-hrGFP (referred to here as pGFP, from Stratagene). pGFP plasmid was propagated in *E. coli* strain DH5α and isolated using Plasmid Maxi kits (Qiagen) according to the manufacturer's instructions. Purified plasmid DNA with an A_260_/A_280_ ratio of 1.8 was used for transfection.

### 2.8. Cell culture

Human embryonic kidney cells (HEK 293T) and human bone marrow mesenchymal stem cells (hMSCs) were grown in DMEM-Glutamax (Gibco) with 10% heat-inactivated FBS (HyClone). Cells were routinely maintained on plastic tissue culture flasks and plates (Sarstedt) at 37 °C in a humidified atmosphere containing 5% CO_2_/95% air. Adult human bone marrow was harvested from routine surgical procedures (pelvic osteotomies) with informed consent, diluted 2-fold in phosphate-buffered saline (PBS) and separated by centrifugation on a Ficoll-Paque layer. After centrifugation at 300 g for 30 min, the mononuclear cell layer was recovered from the gradient interface and washed with PBS. The cells were centrifuged at 150 g for 10 min and resuspended in complete culture medium. Mononuclear cells were seeded on plastic tissue culture flasks in concentration 0.5–1 mln cells/cm^2^. The established primary hMSC cultures were washed 72 hours later and propagated until reaching 75–80% confluence with medium exchange twice a week. The hMSC phenotype was confirmed using flow cytometry analysis with antibodies to CD90 and CD105 (positive), and CD34 and CD45 (negative), using a FACScan analytical flow cytometer (Becton Dickinson).

### 2.9. Transfection experiments

HEK 293T cells were seeded (3 × 10^4^ /well) in 24-well plates in 1 mL of medium. hMSCs (5 × 10^4^ cells/well) were seeded in 6-well plates in 3 mL of medium. All cells were allowed to grow to 65–70% confluence for 2–3 days before transfection. For HEK 293T transfection, complexes of plasmid DNA (2 μg) and P4 dendrimer at a charge ratio of 1:1 were prepared in 100 μL 150 mM NaCl and the samples were incubated for 15 min at room temperature. The time of transfection was two hours. For the hMSC wells, 10 μg plasmid DNA was diluted in 200 μL 150 mM NaCl. The medium was replaced with FBS-free medium before transfection. Following 2 h treatment of the DNA-dendrimer complexes, the medium was replaced with DMEM-Glutamax (Gibco) containing 10% heat-inactivated FBS. hrGFP fluorescence was monitored using microscopy, and the percentage GFP-positive cells were determined after fixation with 2% paraformaldehyde using a FACS-scan analytical flow cytometer (Becton Dickinson).

### 2.10. Cancer cultures

The bioptates from craniospinal cancer of the fourth ventricle (IV stage) were obtained from patient Z and cultivated. The bioptates were washed free of blood and mechanically dispersed in Hanks solution (Sigma-Aldrich, USA) with added gentamycin sulfate. They were placed in solution containing 0.25% trypsin in EDTA (2 mL) for 30 min. Trypsin action was inhibited by the addition of 3 mL fetal calf serum (FCS) (Sigma-Aldrich, USA) CIIIA) for 3–5 min. Material was mechanically dispersed under a microscope and added to Dulbecco's Modified Eagle's Medium (DMEM) (Sigma-Aldrich, USA) containing FCS (1:10) and 4% gentamycin sulfate (10^−4^ g per liter). The cells obtained were cultivated in the medium indicated below for 2–7 days at 37 °C, 95% humidity and 5% partial pressure of CO_2_. After indicated periods the anti-neoplastic drugs and/or dendrimer were added to the center of 2 mL Petri dishes at a recommended dosage recalculated per dish squire (10 cm^2^).

### 2.11. Statistics

Results are presented as mean ± SD (standard deviation), n = 6. Data were analyzed using Student-Fisher test and one-way analysis of variance (ANOVA) with a posthoc Newman-Keuls test.

## 3. Results and Discussion

As part of the current research, the possibility of P4 phosphorus-containing dendrimers binding to the anionic fluorescent probe ANS was investigated.

### 3.1. Binding of fluorescent probe ANS by P4 dendrimer

The fluorescence titration technique is widely used in experimental and clinical studies as a model of interaction between albumin and ligands (bilirubin, fatty acids, hormones, drugs and herbicides) including various diseases [12,14-16]. If the binding centers of albumin are occupied by ligands the capacity of albumin to bind the fluorescent probe decreases. The albumin capacity of binding anionic ligands was measured by different fluorescent probes (ANS, K-35, Pyrrone Red) [12,14-16]. Double fluorimetric titration method was employed to estimate a binding constant and the number of binding centers of P4 dendrimer.

Pure ANS probe in aqueous solution had weak fluorescence in the range 400–600 nm, with maximum fluorescence at 520 nm, and this was a consequence of its low fluorescence yield in a polar environment [11]. Adding P4 dendrimer led to a sharp increase in fluorescence intensity (Figure 1A) and the blue shift of the position of emission maximum (λ_max_) (data not presented). The blue shift of fluorescence emission spectra and the increase in fluorescence intensity in the presence of serum albumin are known to occur for solvatochromic fluorescent probes (*i.e*. ANS) and are the characteristic features of their binding by albumin molecules [11-13]. Therefore, it can be concluded that the observed results were the consequence of binding between ANS and P4 dendrimers. The binding constant (*K*_b_) and the number of binding centers per one molecule (*n*) of P4 dendrimer can be determined using the double fluorometric titration method according to Eqs. (1–6). For this scenario, the double fluorimetric titration ([P4] was set to constant [ANS] and [ANS] to constant [P4]) (Figure 1 A and B).

**Figure 2.**
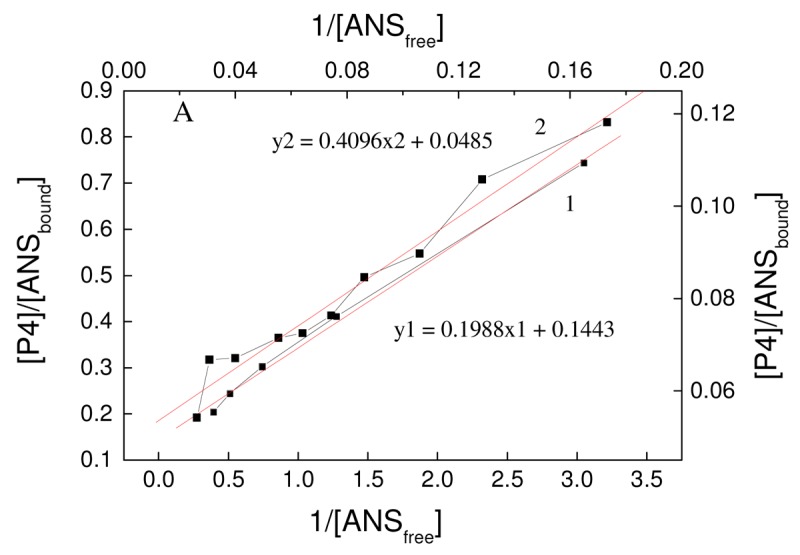
Presence of two binding centers in Scatchard-Klotz coordinates.

Figure 2 presents the binding curves of ANS to P4 in coordinates of Scatchard-Klotz. According to these curves, two binding centers for ANS interacting with P4 were identified with binding constants and the number of binding centers per one molecule of P4: *K*_b1_ = 1.4 ± 0.3 × 10^6^, *n*_1_ = 5 ± 1, *K*_b2_ = 8.4 ± 0.3 × 10^6^, *n*_2_ = 2.4 ± 1.2. The results suggest that one binding site is located deep in the dendrimer, whereas the other is close to or on the surface. If true, these two places where ANS is located would be characterized by different degrees of hydrophobicity. As follows from these data, P4 had binding constants to ANS that were comparable with human serum albumin [14]. Therefore, these features of P4 in terms of a binding system make it comparable to serum albumins. As is the case for albumins, the P4 could bind endogenous and exogenous ligands (*i.e.*, toxins) in blood and may have potential as a detoxicant.

### 3.2. The formation of dendriplex between P4 and anti-HIV siRNA siP24

**Figure 3.**
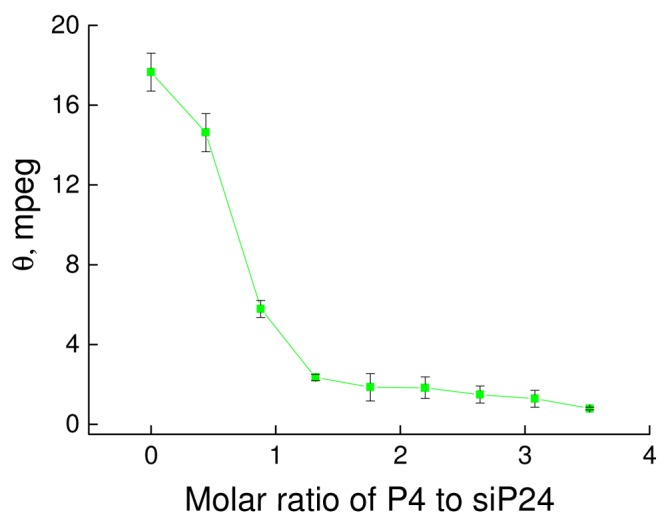
Changes in molar ellipticity of siP24 upon addition of P4 dendrimer. λ_max._ = 262 nm. [siP24] = 10 μM.

For analysis of binding between anti-HIV siRNA siP24 and phosphorus-containing dendrimer P4 the CD spectra of siP24 was measured in the absence and presence of various concentrations of P4 (data not presented), and the molar ellipticity of siP24 was estimated (Figure 3). The molar ellipticity of siP24 decreased sharply to zero upon addition of P4 dendrimer at a molar ratio of ~1.3–1.7. This indicates the formation of a complex between siP24 and P4 at this molar ratio.

**Figure 4.**
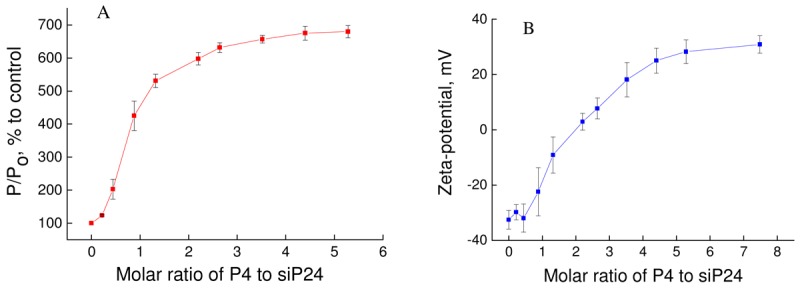
**(a)** Changes in fluorescence polarization of fluorescein labeled siP24 upon addition of various concentrations of P4. **(b)** Changes in zeta-potential of siP24 upon addition of various concentrations of P4. A: [siP24] = 0.1 μmol/L. B: [siP24] = 0.25 μmol/L.

The formation of dendriplex was confirmed using fluorescence polarization and zeta-potential (Figure 4).

The addition of P4 led to sharp changes in fluorescence polarization and the zeta-potential of siP24 up to a molar ratio of three for fluorescence polarization and 4.5 for zeta-potential. These data indicate that formation of a dendriplex between P4 and siP24 occurs between 3 and 4.5 using these techniques. The differences in results concerning CD, fluorescence polarization and zeta-potential can be explained by peculiarities of the techniques employed and the various concentrations of siP24 used.

**Figure 5.**
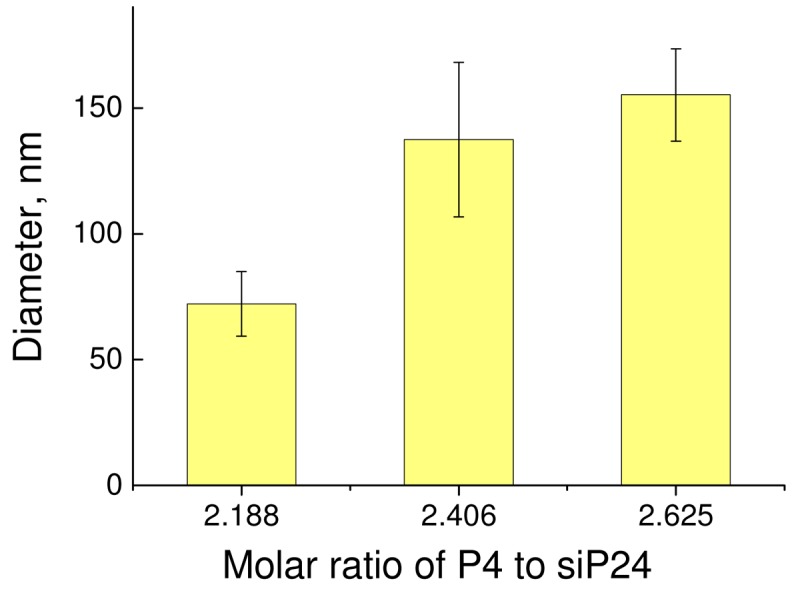
The zeta-size of the dendriplex between P4 and siP24.

The size of dendriplexes was estimated using dynamic light scattering. The molar ratio was used as the top weighted for results obtained from CD, fluorescence polarization and zeta-potential measurements. The results are presented in Figure 5.

The analysis of the zeta-size of the dendriplex between P4 and siP24 at various molar ratios (data not presented) demonstrated that the size distribution of each sample was monodispersed and no particles more than 500 nm were detected.

**Figure 6.**
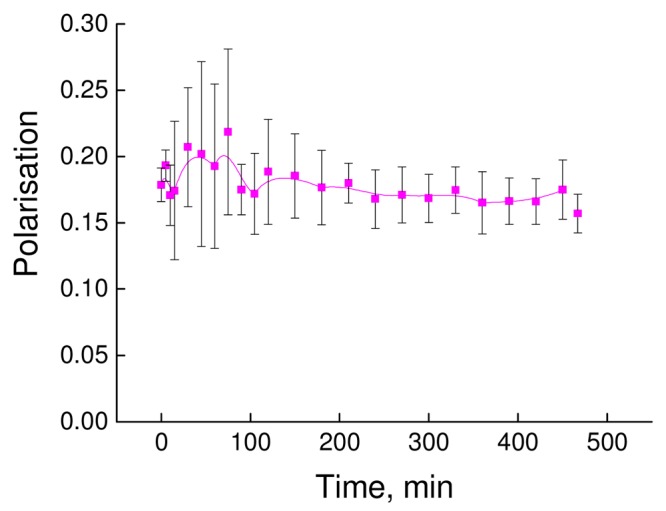
The dependence of the fluorescence polarization degree of the dendriplex formed by fluorescein-labeled siP24 and P4 on the time of incubation.

The stability of the dendriplex in terms of time of incubation was analyzed using a fluorescence polarization technique. As follows from Figure 4A, the formation of a dendriplex led to a seven-fold increase in the degree of fluorescence polarization of fluorescein-labeled siP24 (from ~0.03 to 0.18–0.21). Figure 6 presents the changes in polarization degree of the dendriplex according to time of incubation.

The absence of a decrease in the fluorescence polarization degree of the dendriplex indicates stability of the dendriplex over a period of 25 hours.

Therefore, the results demonstrate that fourth generation phosphorus-containing dendrimers can interact with siRNA and form a stable dendriplex in a charge ratio of (2–3) with diameter of 75–150 nm.

### 3.3. Transfection and cytotoxicity of P4

The efficiency of transfection of the pGFP vector using P4 dendrimer was analyzed in HEK293 T cells and in mesenchymal stem cells. The pGFP gene is frequently used as a reporter of expression [17].

**Figure 7.**
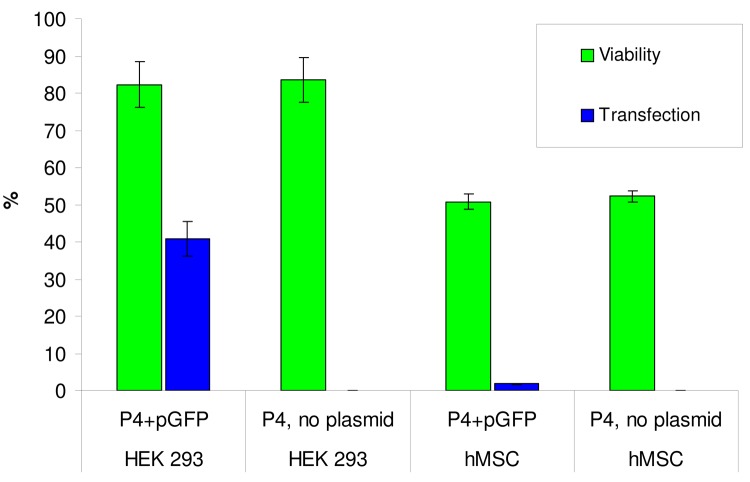
Transfection rate and cell viability parameters for P4 dendrimer in HEK 293 cells and hMSC.

Figure 7 demonstrates the rate of transfection of dendriplex P4-pGFP in HEK 293T cells. The rate of transfection is comparatively high −47.00 ± 5.8%. The mean fluorescence intensity (MFI) of the transfected cells, indicating the level of expression of the reporter gene GFP, was 4853 ± 533. The transfection efficiency for PAMAM G3, G4, G5, G6, G4-OH, G4-25% and G4-50% were investigated (PAMAM G4-25% has 75% NH_3_^+^ groups and 25% CH_3_ groups; PAMAM G4-50% has 50% NH_3_^+^ groups and 50% CH_3_ groups). PAMAM G4 produced the highest level of transfection, reaching 78 ± 6.8% of cells. PAMAM G3 transfected 41.7 ± 5.6% of cells, PAMAM G5 25.69 ± 3.8%, PAMAM G6 13.1 ± 0.78%, and PAMAM G4-25% 0.1 ± 0.7%. PAMAM G4-OH showed zero transfection efficiency. Unexpectedly, PAMAM G4-50% had a transfection efficiency of 20.00 ± 3.8%. The comparison between PAMAM dendrimers and P4 demonstrates that P4 had the highest transfection among these dendrimers, with the exception of PAMAM G4 dendrimer [18,19]. However, the MFI for P4 dendrimer was significantly higher than for PAMAM G4 dendrimer [18,19]. The transfection effectiveness can be influenced both by the dendrimer surface composition and topology, which may affect the dendriplex entry into the cell, and by differences in protonation which define the rate of DNA release. The relative contribution of these and other factors are yet to be determined. The moderate effect may be ascribed to the strong stabilization of the P4 dendrimer over the plasmid that does not allow effective release of the cargo. The difference between PAMAM and P4 is that in the later, there is a net positive charge on the amine groups (terminal amine in the case of P4) while in the case of PAMAM there is no protonation of all amine groups which may reduce the interaction. Zinselmeyer *et al.* [20] reported that DNA was fully condensed by higher generations of polypropyleneimine dendrimers (3rd–5th generations) and only partially condensed by lower generations (1st and 2nd); the lower generations were more efficient at gene transfection. Svenson and Tomalia reasoned that the nano-container properties of a dendrimer depended on the ratio between size and flexibility [21]. Bielinska *et al.* [22] reported increased aggregation of DNA complexes with higher generations of PAMAM dendrimers. Tang *et al.* [23] studied fractionated dendrimers and demonstrated that dendrimers with more flexible branches had greater transfection efficiency. On the basis of these data, the hypothesis was proposed that high generations of dendrimers lose transfection efficiency as their surface charges are too densely packed, and more flexible branches result in greater transfection efficiency. It is possible that the fourth generation of PAMAM and phosphorus dendrimers is optimal for maximal transfection efficiency.

Cytotoxic and genotoxic effects of dendrimer transfection were examined using the MTT assay. Exposing HEK cells to pure P4 demonstrates that DNA removal has no effect on cytotoxicity. As follows from Figure 7, the cytotoxicity of P4 in HEK 293T cells is comparatively low; cell viability without and with plasmid is approximately 82%. The comparison of viability of HEK 293T cells between traditional lineal polyethyleneimine (PEI) and dendrimers demonstrates the advantage of dendrimers. Linear PEI + pDNA 1250 ng/mL results in 16% viability of HEK 293 cells [24], PEI 40–60 μg/mL in viability of 5–30% [25-27], and PAMAM G4 60 μg/mL has viability of 60–80% [25-28], phosphorus dendrimer of 1 generation has viability of 80% [28], PPI dendrimers viability of 50–70% [29,30], and modified non-toxic dendrimers viability up to 100% [25,31,32].

Therefore, P4 dendrimer has comparable characteristics with known dendrimers for gene transfection (transfection rate and viability).

MSCs from bone marrow are an accessible population of human adult stem cells with a number of trophic and immunomodulatory properties that are suitable for *in vitro* expansion and re-implantation. The development of a rapid, easy and safe protocol for plasmid-based gene expression in this type of cell may enhance their potential and facilitate cell-based gene therapy using MSCs.

The transfection rate of P4 dendrimer for mesenchymal stem cells did not exceed 2%. This rate was higher than for PAMAM G3, G5, G6, G4-OH, G4-25% and G4-50% dendrimers (0–0.2%) [18,19] but three times lower than for PAMAM G4 dendrimer (7%). The viability of MSCs upon addition of P4 ranged between 50–55%. Under the same conditions, the viability of PAMAM G4 dendrimer was 90%. Transfection of MSCs using pGFP-dendrimer overnight caused the viability of cells to decrease to 50–52% for PAMAM G4- pGFP dendriplex and to 32–38% for P4-pGFP dendriplex.

As follows from Figure 7, hMSCs are more sensitive to dendrimer exposure than HEK cells. Experiments with removed DNA confirm that the observed decreases in cell viability in both cell types is solely due to dendrimer cytotoxicity, not genotoxic effects of GFP over-expression. Cell viability decreases in the overnight transfection experiment compared to 2 hours exposure time. Cytotoxicity was not significantly different between dendrimers and dendriplexes in this study.

### 3.4. Enhancement of action of anti-cancer drug cisplatin by P4 dendrimer

The low efficiency of treatment of brain tumors is predominantly due to their close proximity to vital centers. Courses of chemotherapy and radiotherapy using protocols HIT`91 and PO/02-PO/04 [33,34] have positive and negative effects.

The negative effects are: (1) low permeability of blood-brain barrier to cytostatics [35,36], (2) low selectivity of courses, (3) open questions in terms of the mechanisms of drug action, making it difficult to estimate the number of molecules interacting with a tumor [37], (4) enhanced toxic effect of courses, leading to destruction of intact cells together with tumor cells and destruction of the immune system [36,38,39].

Due to such negative effects, the possibility of P4 dendrimer being used to enhance the action of the cytostatic drug cisplatin, with the aim of reducing the concentration necessary for the cytostatic effect, was investigated (Table 1).

At high concentrations (10 and 100 μg/mL) P4 has a cytostatic effect that is not present at low concentrations. At a concentration of 1μg/mL, cisplatin can result in cell death.

However, the combination of P4 dendrimer with cisplatin (1 + 0.1 μg/mL) led to the significant death of tumor cells, confirming that P4 enhances the toxic effect of cisplatin. These results are in agreement with other studies concerning the efficacy and efficiency of dendrimers for the delivery of anti-cancer drugs. The main mechanism of such enhancement is the direct delivery of anti-cancer drugs using non-covalent conjugation with dendrimers [40,41]. The difference in the effect induced by the combination of P4 dendrimer with cisplatin 1 + 0.1 μg/mL and 10 + 0.1 μg/mL can be explained probably by a different protection of the drug by the dendrimer. The exact reasons for this effect will be determined in future experiments.

## 4. Conclusions

Normally, introduction of free nucleic acid is accompanied by its enzymatic degradation in the organism. Furthermore, DNA and RNAi effectors are unable to cross biological membranes largely owing to their strong negative charge, inducing poor cellular uptake. Therefore, delivery agents should maintain the biological activity of drugs and promote cell penetrating activities so that the process of DNA and RNAi is efficient; this necessitates the existence of vectors for nucleic acid packing and transport. P4 dendrimers as vectors can effectively bind hydrophobic substances and anti-HIV siRNA, can transfect intracellular DNA and can enhance the action of cytostatics. Therefore, fourth generation cationic phosphorus-containing dendrimers can be good candidates for drug and gene delivery carriers after experiments *in vivo*.

## Figures and Tables

**Figure 1.**
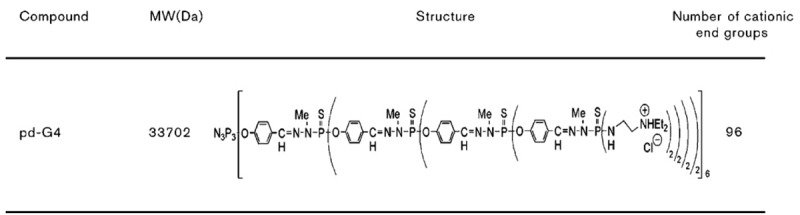
Fourth generation phosphorus-containing dendrimer.

**Figure 1.**
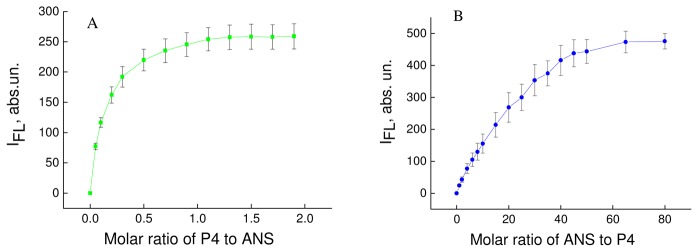
The first (**a**) and second (**b**) fluorimetric titration curves of 8-anilino-1-naphthalenesulfonate (ANS) and P4. **a**: constant concentration of ANS equal to 4 μM. (**b**):constant concentration of P4 equal to 0.5 μM.

**Table 1.** The cytostatic effects of cisplatin and/or P4 dendrimer.

**Serie**	**Concentration (μg/mL)**	**Cytotoxicity, %**
Control	No drugs	42.7 ± 4.2
Cysplatin	1.0	61.9 ± 8.8
P4	100.0	63.5 ± 2.5
P4	10.0	65.5 ± 0.8
P4	1.0	42.9 ± 4.7
P4 + cysplatin	10.0 + 0.1	53.4 ± 8.6
P4 + cysplatin	1.0 + 0.1	74.1 ± 4.1
